# Urban fire risk control: House design, upgrading and replanning

**DOI:** 10.4102/jamba.v10i1.522

**Published:** 2018-05-30

**Authors:** Isabela Wilfred Mtani, Elinorata Celestine Mbuya

**Affiliations:** 1 Institute of Human Settlement Studies, Ardhi University,United Republic of Tanzania

## Abstract

Urbanisation leads to house densification, a phenomenon experienced in both planned and unplanned settlements in cities in developing countries. Such densification limits fire brigade access into settlements, thereby aggravating fire disaster risks. In this article, we assess the fire exposure and risks in residences in informal areas of Mchikichini ward, in Dar es Salaam City, Tanzania. We rely on interviews of residents and government officials to obtain background on the occurrence and causes of fire accidents, policy provisions and regulations, and experiences with fire outbreaks and coping strategies, as well as on observations and measurements of house transformations, spatial quality and indoor real life. Our findings suggest that fire risks arise from both inappropriate structural characteristics and unsound behavioural practices. This includes unsafe electric practices by residents, poor capacity of residents to fight fires once started, limited access to structures by firefighting equipment because of flouting of planning regulations and inadequate awareness of local government leaders of the magnitude of fire risks. Potential changes to reduce fire risks in the settlement include the installation of firefighting systems, restriction of cooking to designated spaces, use of safer cooking energy sources and lighting means, improvements of vehicle access routes to neighbourhoods, capacity building at the grass root level and the establishment of community-based fire risk management.

## Introduction

A disaster results from an extreme event that exceeds the capacity of the affected area to respond with measures to save lives, preserve properties and/or maintain its social, ecological and economic stability (Pearce 2000). The impacts of natural disasters have risen over recent decades, affecting social development and economic and political activities in developed and developing countries alike (Munasinghe 2014). These impacts include a range of direct, indirect and secondary effects, and can be both tangible and intangible. Direct impacts relate to the physical damage people may suffer, such as disabilities, psychological harm, interrupted social services, loss of property and means of livelihood, and relocation, while indirect impacts reflect the burden put by displaced residents on friends and relatives who feel obligated to help.

Hazards comprise events that result in harmful potential consequences such as injury, loss of life and damage to property or environment (Cambridge English Dictionary 2017). They include both man-made and technological events (EU 2016). An example of the former could be development of housing in a neighbourhood that leads to blockage of roads by which firefighting equipment can access fires. In the same neighbourhood, a technological hazard could be something as simple as unsafe electrical practices in a home.

The relationship between disaster risk reduction (DRR) and sustainable development is globally recognised in the Hyogo Framework for Action 2005–2015, adopted in 2005. In this framework, DRR is a crosscutting concern that affects sustainable development at all levels. More broadly, the Millennium Development Goals established following the 2000 United Nations Millennium Summit recognise the importance of safety and resilience in cities, towns and human settlements, which includes safety against fire risks and disasters. These objectives are re-acknowledged under Goal 11 of the Sustainable Development Goals (SDGs), which aims at making cities and human settlements inclusive, safe, resilient and sustainable (United Nations 2015). The principles of DRR suggest that in any disaster environment, the most important takeaway is that the extent of damage, destruction and casualties during fire outbreak largely depends on the level of exposure and vulnerability of the settlement itself. These can be heavily influenced by the nature of the settlement – whether it has been formally planned or has arisen as an informal settlement without official sanction – and the level of resources available to its residents.

This study analyses the extent of fire disaster risks in informal settlement, using a case from the city of Dar es Salaam, Tanzania. We aim both to understand the special fire risks in informal settlement settings and to explain the community’s capacity to reduce these risks through planning and implementation of urban fire risks control. We focus particularly on accessibility by fire tenders, house types and designs, and cooking energy regarding their contribution to increased cases of fire risks in the settlement.

### Background

Typical causes of fire disasters in urban areas include usage of wood fuel and charcoal for room heating, poverty, ignorance, waste burning around courtyards (Ibrahim & Mussa 2011); poor city infrastructure for firefighting and accessibility (Gernay et al. 2016); and insufficiencies in disaster prevention policies (Voogd 2004). Frequent fire disasters are often common in crowded central business districts (CBDs) and markets (Navitas 2014; Oladokun & Emmanuel 2014; Voogd 2004) and are one of the major concerns for urban planners. Congestion and high density of buildings and the people increase the potential for fire risk occurrences, heightening the fire risks and rescue efforts.

An assessment of the likelihood of occurrence of potential hazards in Tanzania confirmed fire hazards as one among those hazards with a high likelihood of occurrence, but only a moderate impact on public health and safety. Conversely, the estimated impact on property because of possible fire outbreaks ranks high (URT 2012). Kihila (2017) and Kachenje, Kihila and Nguluma (2010) noted numerous reasons for high fire risks in buildings in Tanzania, including non-functioning firefighting facilities installed in buildings, lack of familiarity with firefighting equipment operations, unawareness of fire escape routes, inaccessibility to residences by the firefighting equipment, poor communication links that delay fire brigade assistance, inadequate means and facilities for firefighting, insufficient water to run firefighting equipment and a lack of community knowledge and awareness on the extent of fire risks.

The firefighting framework and protocol stipulated by the Prime Minister’s Office, Disaster Management Department through its emergency preparedness and response plan provides operational concepts, organisational responsibilities and guidelines for direction and control during fire outbreaks. Nevertheless, this framework remains silent about provisions to address challenging informal settlement characteristics of cities and towns in Tanzania, such as narrow passages and difficulties in accessing houses and other building structures from streets.

The primary agency authorised for firefighting in Tanzania is the Department of Fire and Rescue Force, which is mandated to plan and support standard operating procedures. Supporting agencies include the Tanzania Police Force, Tanzania People’s Defence Force, Department of Emergency Preparedness and Response Section, Tanzania Ports Authority, Tanzania Civil Aviation Authority, Department of Water Resources and Tanzania Electric Supply Company (TANESCO). Tasks assigned to the public agencies include fire detection, fire control and evacuation support. In addition, private companies may assist in firefighting during disasters at the discretion of the Commissioner General of Fire and Rescue Force (URT 2012).

The *Fire and Rescue Act* of 2007 imposes fire-related requirement on buildings depending on their characteristics. For example, buildings that are at least four storeys high have to be provided with adequate firefighting means and facilities. Even with such facilities, however, many of these buildings remain at high fire risk because the majority of occupants residing or working in the buildings do not know how to operate the facilities and/or are unaware of the available escape means in case of fire outbreak (Kachenje et al. 2010). Moreover, in many informally settled areas, these regulations may be unenforced, and housing development within them has encroached on the access roads. Reclaiming these roads is crucial for fire risk reduction. Replanning processes to reduce fire risks have seen some success elsewhere, such as in Bondeni settlement in Voi, Kenya, which lies 150 km northwest of Mombasa. Bondeni has existed as a squatter community since 1950s, but replanning and upgrading in 1991 led to improved houses, infrastructures and sanitation systems in the settlement. The improvements reduced fire risks to dwellers by improving accessibility through provision of a 12-m-wide road and network of bitumen felt (Mwangi 1997).

### The settlement

One of the urban settlements in Dar es Salaam, which experiences frequent fire outbreak, is Mchikichini ward.

**FIGURE 1:**
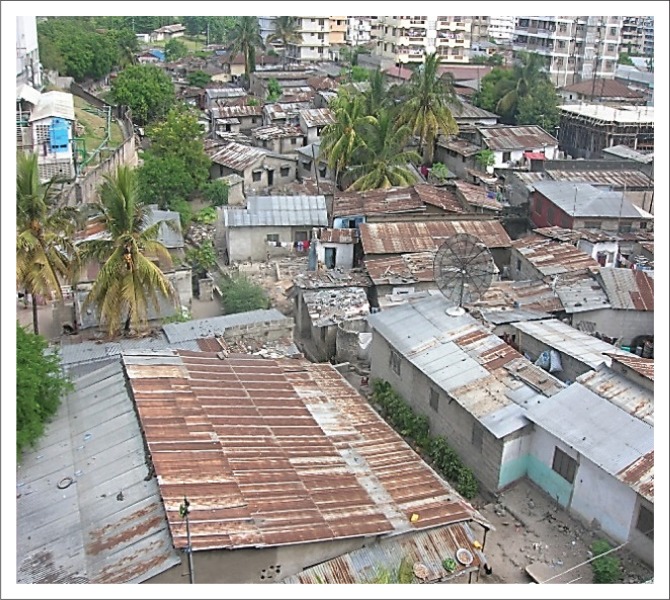
Aerial view of Mchikichini ward.

Mchikichini is an administrative ward in Ilala District in southwestern Dar es Salaam, situated about three kilometres from the central business districts of Kariakoo and the city centre and thus readily accessible to them by low-cost public transport. The settlement, which housed roughly 20 000 inhabitants in 2002,^1^ has continued to grow from an influx of rural residents into Dar es Salaam in search of employment opportunities and exceeds 25 000 as of the recent national census in 2012 (URT 2012). The average household size is five people and the total residential area is 2036 hectares (Msilu 2009). The settlement’s population growth has led to rapid development of informal housing, and infrastructure services have not kept up. In addition to the population influx, the higher house density and higher household sizes typical in informal settlements compared to formally planned areas (Makoba 2008) have yielded a population density of 15 139/km^2^ (URT 2012), resulting in overcrowded housing environment (Figure 1). House densification over the years has grown from a mere six houses/ha in the mid-1980s to 23 houses/ha in the mid-1990s to 27 houses/ha in the early 2000s to 33 houses/ha in 2012 (URT 2012), with preliminary information suggesting house density at the time of this writing at 48 houses/ha. Because house densification has come through continuous, spontaneous growth rather than as a result of planned process, the small patches of land that existed decades ago are presently covered with buildings.

**FIGURE 2:**
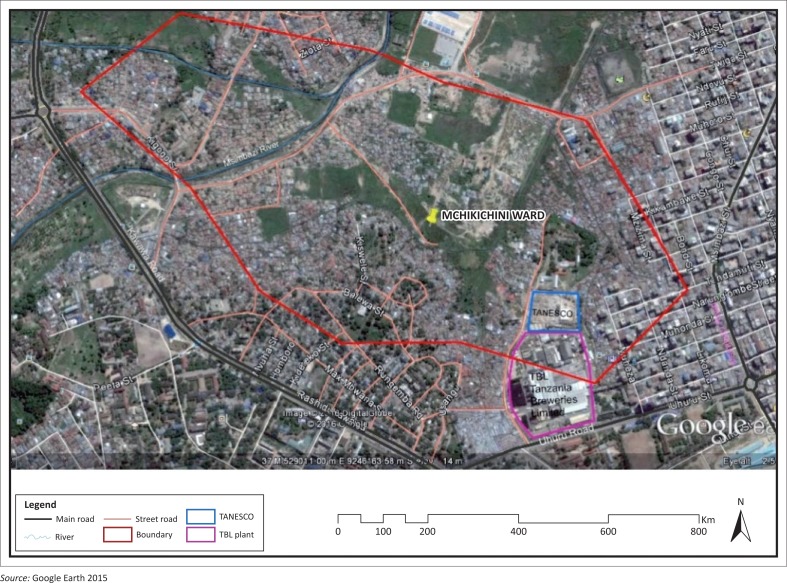
Accessibility and physical development in Mchikichini settlement.

The major reason of attraction to this settlement is its strategic location to the Dar es Salaam CBDs of Kariakoo and the city centre, which makes it a proper living area for the majority who save on daily transport cost to and from their homes in Mchikichini to the CBDs. The ward has both planned and unplanned areas, with approximately 50% of Mchikichini residents living in the latter (Dar Ramani Huria 2016). Poverty has contributed to densification of this unplanned part as land owners needing more space lack enough income to afford land elsewhere and hence extend their houses into the rights of way nominally reserved for vehicular access. In informal areas, such rights of way are normally allocated by mutual understanding between house owners, allowing limited means to claim or enforce them more formally. In addition, the means of access from the outside into the Mchikichini settlement comprise a network of streets and footpaths of uneven quality. The settlement lies in close proximity to Uhuru Road and Msimbazi Road, both of which are major thoroughfares. While major roads are tarmac level and generally in good condition, all other road and footpath access is through earth roads and unconstructed footpaths (Figure 2).

Despite much of it being unplanned, Mchikichini has a variety of thriving public and private institutions, including TANESCO, Tanzania Breweries Limited and Karume, a market selling second-hand items. In addition, adjacent to the settlement, the newly constructed Machinga complex employs thousands of petty traders, further attracting settlers who want to live adjacent to their livelihoods. The majority of Mchikichini’s inhabitants remain low-income earners (URT 2012), though, even with the unusually close access to the employment opportunities afforded by the CBDs and Machinga complex. Roughly 75% of its households are self-employed in the informal sector, with most of the activities being home based or undertaken along streets and roads. Common livelihood activities that provide a living for the majority of Mchikichini residents include food vending, selling fresh vegetables and fruits, operating retail shops, selling new and used clothing and garments, carpentry and unisex hair care salons. Majority of inhabitants are low-income earners, while few households are middle-income earners (URT 2012), even though the Vice President’s office argues that generally poverty remains a rural phenomenon (REPOA 2012; URT 2012). The National Strategy for Growth and Poverty Reduction (MKUKUTA) report indicates a steady growth of gross domestic product (GDP) from 4.1% in 1998 to 7.4% in 2008, with the highest growth in 2004 at 7.8%. The GDP grew to 6.9% in 2012 (NBS 2012). The report acknowledges that in spite of the sustained growth rates, a slight reduction of 2% in household income poverty has been achieved over the same period in urban centres.

### Conceptual framework

Our study’s framework utilises concepts of disaster risk management and its interaction with urban planning. We build on the premise that the degree of compliance with planning guidelines determines fire exposure, which in turn determines risk. While a settlement’s planning limitations clearly shape its exposure to fire accidents, several other design factors link either directly or indirectly to form or eliminate fire risk exposure in any given area. For instance, the layout of buildings in terms of space functions may lead to fire eruption and accidents, and the design and installation of services such as through electricity connection codes and policies may influence fire risk.

Increasing densification of buildings, an urban planning issue, contributes to urban fire exposure, which is a particularly common urban dynamic in developing countries where informal settlements are rapidly forming. Socio-economic variables may be equally important. These include household financial resources, which influence the type and safety of energy used for cooking and lighting, and the night cooking practices. Attitudes and perceptions towards fire risk also form a crucial input in the framework, as do the roles and responsibilities of public and private actors. Often an unaccountability gap in responsibility forms between actors, especially between house developers and technocrats at the municipal and central government levels (Wamsler 2006). It is important to document the perceptions of all of these actors because home developers and occupants often see themselves as victims of fire disasters, ignoring their own contributions to risk outcomes and allocating all responsibilities for prevention to the municipality (Wamsler 2006).

In conceptualising fire risk management, the magnitudes of fire accidents necessitate rethinking the possible social, economic and emotional factors that contribute to risk and the impacts that could result. Among critical issues is the role of policies and regulations in ensuring fire safety to urban residents in informal settlements.

## Methodology

We adopted a case study strategy to examine fire risk in informal settlements because this issue is contemporary and evolving and requires a rich information case. We undertook a variety of both qualitative and quantitative approaches:

- Focus group discussions and stakeholder meetings at the beginning of the study to construct an overview of issues related to fire hazards and risk at different levels and to identify data availability.
- Open-ended questionnaire to provide information about ownership and tenure, experiences with fire outbreaks, emotional, social and economic impacts suffered and measures taken by the family to prevent or cope with fire outbreaks.
- Literature review to unearth important concepts, including practices from other countries, to guide the study and confirm evidence from other sources.
- Measurements of physical aspects such as functional spaces in houses and widths of streets and passages, which support our analysis of space use and recommendations for improving road access.
- Observations on the extent of house extensions blocking access roads and cooking practices of study participants to assess fire risk and community awareness (Sekizawa 2005).
- Photographs too were systematically taken to document relevant indoor real-life situations and outdoor spatial qualities, particularly in relation to congestion of houses and accessibility.

Key informant interviews of actors in relevant institutions to get in-depth information of policy provisions, by-laws and other requirements in relation to the study subject, as well as cooking behaviour and practices for coping with fires, were also conducted. The institutes included the Disaster Management Training Centre (DMTC) of Ardhi University; Department of Fire and Rescue Service Force; Ilala Municipal Council; Mchikichini Ward Office; Police Force – Msimbazi Central Police; Dar es Salaam Water and Sewerage Corporation (DAWASCO); and the East African Breweries Limited which occasionally provided support and fire tenders in firefighting upon occurrences in the settlements. The interview also focused on households’ practices related to decisions on energy type used, particularly for cooking, and other open-flame sources.

Because of the disorganised nature of the settlements, we found it difficult to pre-identify houses for interview. Rather, we identified houses *in situ* during the data collection exercise. Stratified random sampling was done out of a population of 976 households, where 110 households were sampled for an in-depth household survey, representing a 95% confidence level. Where household heads could not be accessed, sampling was objectively done focussing on adjacent houses where information was obtained, ensuring that the research protocol was complied with.

Data analysis included both descriptive analysis to discern patterns, their meaning and implications and impacts to policy on urban planning and fire risks reduction, and simple frequency and graphical analysis of our questionnaire results.

## Findings and discussion

Preliminary investigation through a rapid appraisal of Mchikichini shows 68% of households are aware of fire risks in their settlement. This partly reflects the influence of an awareness campaign conducted by the central government’s Department of Fire and Rescue Force, whereby 26% of the settlers received training on the level of fire risk exposure they are in, as well as the causes of this exposure. It also reflects live experiences, as 46% of residents of Mchikichini have either witnessed or personally experienced fire accidents in the area.

Our research suggests limited fire risk control capacity in residential buildings in the settlement of Mchikichini, as indicated by lacking of coping measures. Reflecting on the urban planning perspective, there appears to be inadequate engagement and contemplation of urban planning concepts, a common issue in informal urban settings of developing countries (Freire 2006); that is, Mchikichini has not integrated urban planning principles into informal settlements. Such principles would require an allowance for appropriate passage of people and fire tenders where informally settled areas are already saturated, as well as implement building regulations on fire control in residential buildings. The community assistance and collaboration is a useful intervention during fire outbreak; however, passage of the people from one point of the settlement carrying water and sand buckets is obstructed by congested houses and narrow or absence of passages. Informality is largely left as an unattended challenge, meaning that urban planning and design interventions to reduce risk are not optimally employed. In addition, even the enforcement of building regulations does not enhance fire safety for houses because such regulations are inadequate for fire safety protection as they do not provide for informal settlements.

**FIGURE 3:**
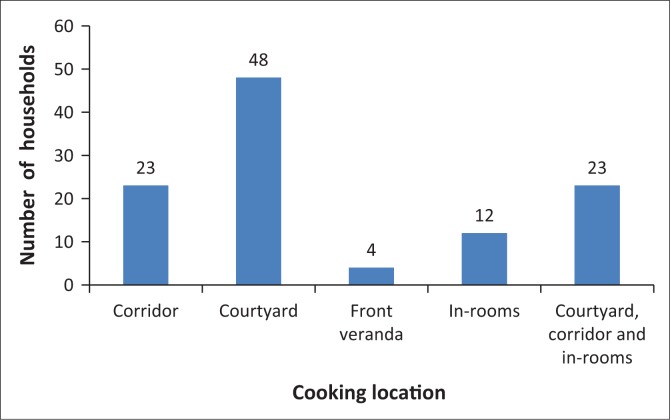
Cooking locations in Mchikichini settlement.

**FIGURE 4:**
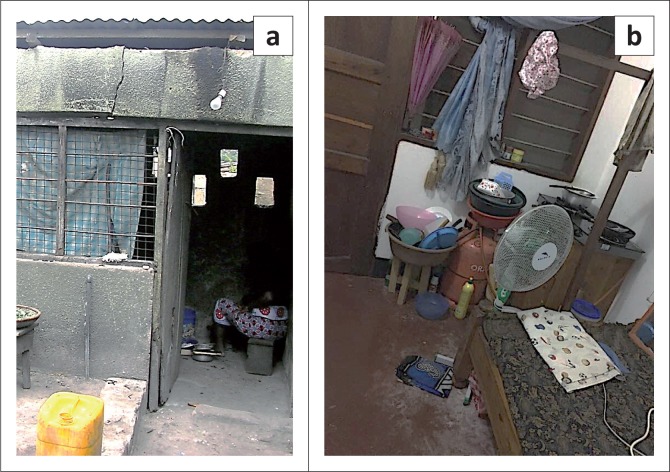
(a) Indoor cooking in a confined space increasing fire exposure. (b) Indoor cooking in undesignated and congested space increasing fire exposure.

We also find that unsafe cooking practices cause frequent fire outbreak in Mchikichini. While 44% of households use their courtyard for cooking and nearly 5% the front veranda, 21% of households cook along corridors and 11% of families cook inside rented rooms. These corridors and rooms are used for multiple purposes, including living and sleeping (Figure 3 and Figure 4), and are normally congested and not well ventilated. Flammable materials such as clothes, mattresses and wooden cupboards contribute to the spread of fires.

In addition, we also observe that a day’s activity patterns affect cooking inside rooms. Families who arrive home late resort to cooking inside rooms for safety reasons (few neighbours are outside) and to simultaneously carry out other activities to save time. In addition, the time of the day when cooking is done affects the cooking location, with early mornings, evenings and late nights associated with indoor cooking and afternoon cooking typically done outdoors. As stated by one of the respondents:

‘I live alone. I cannot really cook outside without a companion to sit with. Besides, it is late and I need to prepare for tomorrow so I have chores to wind up in my room.’^2^

**FIGURE 5:**
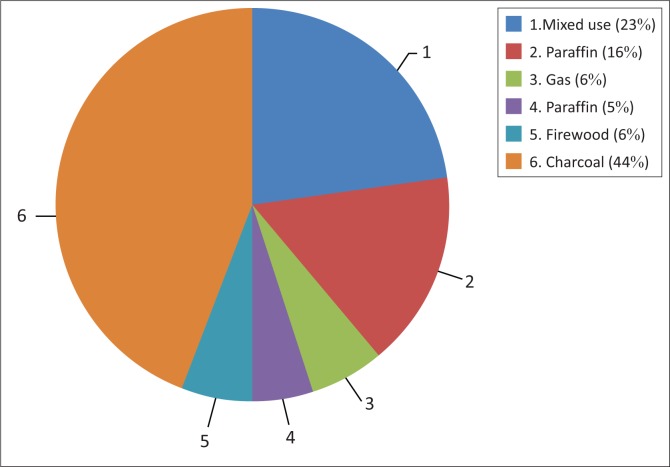
Energy sources in Mchikichini.

A household’s energy source also may contribute to different fire risk levels. Similar to most informal settlements in Dar es Salaam, the majority of residents in Mchikichini’s informal settlements (97%) cannot afford electricity for cooking, and instead rely on paraffin (16%), charcoal (44%) and firewood (6%). Gas cooking, while popular in many parts of the city, only accounts for a 6% share in Mchikichini. In addition, 23% of households use a combination of energy sources, most commonly gas (for quicker cooking, generally in the morning) and charcoal (Figure 5). While 16% of respondents expressed source of energy for cooking was a cause of fire outbreak, 38% of respondents linked fire incidents with cooking location. Other reasons such as lighting sources, for instance, candles, contribute to fire outbreak by 5%. Each energy source exposes the dwelling and its occupants to some levels of fire risks depending on the knowledge of users for a particular energy type, installation and operating technology and cooking location within a dwelling in relation to congestion.

In an informal area such as Mchikichini, risks from using paraffin stoves are heightened because of congestion, high house occupancy rates and poorly ventilated indoor environment. In addition, several health issues come from emissions from paraffin cooking stoves, including respiratory infections, lung cancer and a higher prevalence of asthma (Kankaria, Nongkynrih & Gupta 2004). While Ahrens (2016) reports that households using electric ranges have higher risks of cooking fires and associated losses than do those using gas ranges, the rate of reported fire per household in Mchikichini ward is 3.4 times higher with charcoal than with any other sources of energy. This is because of the charcoal night users usually use indoors.

**FIGURE 6:**
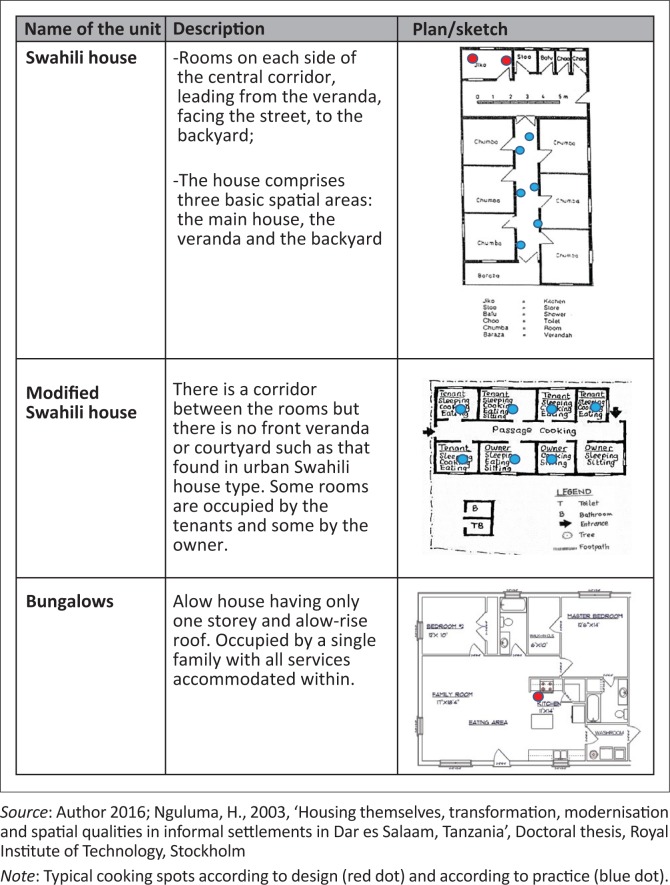
House types found in Mchikichini.

**FIGURE 7:**
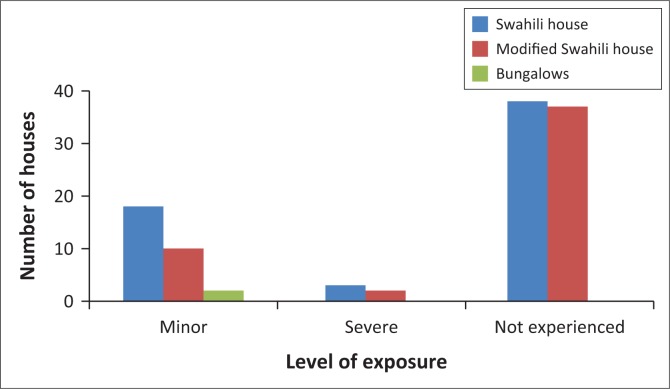
House types relationship with fire exposure.

House types appear to play the most substantial role in creating fire risks as the occurrence of fire hazards relates primarily to the cooking location within a household (see Figure 6 for a description of the most common types in Mchikichini). The majority of houses are either Swahili or modified Swahili types, meaning that cooking is mostly done along corridors or in the courtyard. Our analysis suggests that roughly 16% of Swahili and 9% of modified Swahili house types have experienced minor fire accidents in our study area, and nearly 3% of Swahili and 2% of modified Swahili houses have experienced severe fire accidents. In contrast, none of the bungalows with separate cooking spaces has experienced severe accidents, and only 2% have had minor incidences of fire accidents (Figure 7).

Once a fire starts, accessibility becomes the major issue related to control efforts. The informally settled area of Mchikichini is inaccessible by vehicles, implying that fire tenders cannot reach a particular house experiencing a fire. Ironically, while such vehicles appear generally available, they remain mostly stuck at the border of the settlement near the brewing plant. Given the difficulty of outside access to the area, many residents and the community itself rely on social networks within the settlement for rescue and assistance during fire outbreaks. Residents normally rush to an incident area, irrespective of whether they know the occupants of the building, using sand and water, or tamping with green tree branches, to combat the flames. However, in some places, houses are too tight (60 cm) for a person to pass while carrying tree branches, or buckets of sand or water. At some places, only a single person can pass at a time, with no possibility for two people to pass each other going opposite directions at the same time. Points to draw water for fighting flames also appear lacking within the settlement, even though residents claim such points are located along the main road, which lies only 50 m from the settlement. We could find no evidence of their existence. Moreover, the fire hydrants that existed within the settlement’s boundaries have long been worn out or damaged during house construction and infrastructure upgrading, and no replacements have been provided to date.

The frequency of fires and their associated losses in Mchikichini suggest a need for residents to either change their practices to reduce fire risk or prepare to regularly fight fire outbreaks. However, despite widespread awareness of the vulnerability of the settlement to fire risks, 66% of the households interviewed responded that they take actions only when fire incidents occur. They store no water for firefighting and they do not educate each other on how to reduce fire risks. Similarly, one would expect that in such a risky environment, simple equipment such as hand fire extinguishers would be readily available, but such equipment are completely lacking at the community, *mtaa*, or ward level. None of these echelons has taken the initiative to develop safety guidelines for fire risk, leaving the area with no formalised coping strategies to reduce residents’ vulnerability to fire accidents at either the individual or the community level.

Our study also reveals that 44% of Mchikichini ward residents employ unsafe electric practices (including overloads on sockets and plugs or use of non-specified ones), engage unregistered electrical technicians for installations and repairs contrary to manufacturers’ specifications and use non-standardised electrical appliances with no proper maintenance. Many houseowners also resort to illegal connections to electricity from adjacent or other neighbouring houses, increasing fire risks with unregulated and patchwork electrical installations. This partly reflects the difficulty of overcoming bureaucratic procedures involved in legal transmission from the electricity supply authority to homeowners, as well as the high costs of electricity hook-ups. However, the biggest driver relates to knowledge and understanding of the fire risks. Residents appear generally unaware of the extent of risk these undesired practices impose. In our views, the risk levels are not justifiable by the community on financial, time or other resources required for firefighting. In addition, the degree of congestion of houses adds to the fire risks, particularly when dealing with a utility as dangerous as electricity, if inappropriately handled.

Overall, we find that awareness, understanding and responsiveness to fire risks strongly correlate among the residents we interviewed. Despite explanation by the local government officials that fire risks is an issue of which the Mchikichini community is well aware of, further discussion on awareness reveals contradictions. Roughly three-quarters of our survey respondents claim that they are well aware of the risks associated with fire outbreak, yet most express it as a non-priority concern relative to basic, immediate household needs. The other one-quarter of respondents express a more limited understanding of fire risks and seem generally unaware of fire exposure matters. This group appears to view fire outbreaks as accidental and beyond their ability to control. Leaders at upper levels of administration, even outside the ward, also appear to have inadequate attentiveness to the issue. This represents one of the key challenges for addressing the problem of fire risks.

## Conclusion

The challenge of fire risks in informal settlements is part of a broad developmental context. The exposure to an increasing fire risks in the informal settlements of Mchikichini in Dar es Salaam, Tanzania, is associated with two broad factors: (1) limitations in residential buildings design with respect to cooking spaces and types of cooking energy and (2) limited human and vehicular accessibility within the settlement. The absence of appropriate and continuous planning strategies extends these difficulties, leading to high occupancy rates per house, high house density and closeness of buildings.

Any house type allows specific functions to be carried out in certain functional spaces. House types that allow indoor cooking in a congested room containing flammable materials expose the buildings, occupants and assets to higher risks compared to house types where cooking is done in a formal kitchen space. In Mchikichini’s Swahili houses, where the majority of our respondents live, the study reveals that cooking unfortunately takes place along corridors and in the courtyard. Our empirical evidence shows that these houses experience more fires than bungalows with a separate kitchen. In addition, our study shows that the majority of low-income families in the informal areas rely solely on cheaper and less contained energy sources for their cooking needs, generating a higher exposure to fire risks. The distinctive lack of clarity regarding requirements and responsibilities for fire safety processes, designs and procedures within residential accommodation and other buildings in informal settlements exacerbates this problem.

Fire poses a high risk in dense informal settlements. Disaster management policy has short-changed this risk, partly because of the absence of comprehensive data on fire incidents in such areas. However, our study suggests numerous opportunities for action. These include the development of guidelines for mandatory fire risk assessments and building regulations specific to informally settled areas, improvements in household energy practices in terms of lighting and cooking sources, clarification of landlords’ responsibilities for fire prevention facilities, neighbourhood replanning and upgrading of densified settlements, promotion of community-based strategies on fire outbreak control and management, promotion of coping strategies to fire risk control that show potential, outreach to sensitising the community about the importance of maintaining access roads for fire outbreak control, encouragement of changes in fuel use to adopt safer sources and the establishment of a sub-ward committee for DRR.

## Footnotes

1 2002 Population and Housing Census General Report, Tanzania.

2 An interview with respondent number 4 of Mchikichini who arrives home after 21:00 and does her cooking inside the room.
